# Dr. Levi E. Horton

**Published:** 1912-06

**Authors:** 


Dr. Levi E. Horton of Avoca, N. Y., died April 22, 1912, aged 67. He was a graduate of N. Y. Eclectic, 1891.

